# Facial expression recognition in virtual reality environments: challenges and opportunities

**DOI:** 10.3389/fpsyg.2023.1280136

**Published:** 2023-10-11

**Authors:** Zhihui Zhang, Josep M. Fort, Lluis Giménez Mateu

**Affiliations:** Escola Tècnica Superior d'Arquitectura de Barcelona, Universitat Politècnica de Catalunya, Barcelona, Spain

**Keywords:** virtual reality (VR), facial emotion recognition, VR devices, Quest Pro, Vision Pro, emotion

## Abstract

This study delved into the realm of facial emotion recognition within virtual reality (VR) environments. Using a novel system with MobileNet V2, a lightweight convolutional neural network, we tested emotion detection on 15 university students. High recognition rates were observed for emotions like “Neutral”, “Happiness”, “Sadness”, and “Surprise”. However, the model struggled with 'Anger' and 'Fear', often confusing them with “neutral”. These discrepancies might be attributed to overlapping facial indicators, limited training samples, and the precision of the devices used. Nonetheless, our research underscores the viability of using facial emotion recognition technology in VR and recommends model improvements, the adoption of advanced devices, and a more holistic approach to foster the future development of VR emotion recognition.

## 1. Introduction

The intersections of technology, emotion, and human experience have led to groundbreaking advancements and insights in numerous fields. Two technological paradigms that have witnessed profound evolution over the decades and remain at the forefront of such intersections are Virtual Reality (VR) and Emotion Recognition.

VR technology, from its inception, has promised to revolutionize our perception of reality. Emerging as a game-changer in the world of interactive entertainment, VR offered players not just games but lived experiences. The watershed moment for this was marked by the release of devices like the Oculus Rift, signaling a surge of interest and investment in the VR domain (Tan et al., 2015). However, the realm of VR isn't confined solely to gaming. It spans diverse genres, from adventurous explorations to competitive sports, and with the continuous strides in AI and interactivity, the horizon looks even more promising for VR gaming.

Beyond entertainment, VR's tentacles have reached out into transformative areas such as education, where it replicates real-world scenarios, affording students hands-on opportunities (Merchant et al., 2014). The medical sector has not been untouched either. Breakthroughs in patient rehabilitation and surgical simulations have been made possible, offering new avenues for practitioners (Rose et al., 2005). The immersive nature of VR has found resonance in architectural design, letting clients and designers visualize spaces before they materialize (Wang et al., 2013). Furthermore, in a world challenged by a pandemic, virtual tourism stood out as a beacon for the travel industry (Guttentag, 2010).

VR's influence on psychology is particularly compelling. By generating controlled environments, clinicians have found success in treating conditions ranging from anxiety and phobias to pain management, assisting countless individuals to overcome their fears and traumas (Hoffman et al., 2000; Gorini et al., 2010). Moreover, VR environments have proven especially useful for children with autism, enabling them to practice social scenarios and hone their interpersonal skills (Parsons and Mitchell, 2002).

Parallelly, Emotion Recognition technology has undergone transformative phases, evolving from rudimentary self-reporting methods, which were often influenced by personal biases and cultural nuances (Mesquita and Frijda, 1992; Russell, 1994), to sophisticated deep learning models. The journey has seen the incorporation of biopsychological insights, with physiological signals like heart rate and skin conductivity becoming tools for more precise emotion detection (Picard et al., 2001; Koelstra et al., 2012). The theories of eminent scholars like Ekman gave impetus to recognizing emotions through facial expressions (Ekman and Friesen, 1978; Pantic and Rothkrantz, 2000), while the tonal nuances of voice also emerged as vital emotional indicators (Corive et al., 2001; Scherer, 2003).

The digital age supercharged the field of Emotion Recognition. With machine learning techniques, textual data became a source of sentiment analysis, widely used for gauging public opinions and gathering customer feedback (Liu et al., 2005; Pang and Lee, 2008). The complex architectures of neural networks, such as CNNs and RNNs, provided unprecedented precision in processing multi-modal emotional data (Tzirakis et al., 2017).

Where the worlds of VR and Emotion Recognition converge, we witness a magnified potential for insights. This convergence has enabled researchers to understand and assess “presence” or “immersion” within VR platforms, thereby amplifying the intensity of emotional responses (Riva, 2005; Slater, 2018). Combining VR with traditional self-reporting methodologies has further refined our understanding of emotions in simulated real-world scenarios (MacCann et al., 2003; Felnhofer et al., 2015). The incorporation of biometrics such as EEG and ECG into VR frameworks offers a richer, multi-dimensional view of emotional responses (Mingyu et al., 2006; Ito et al., 2017; Tauscher et al., 2019). Moreover, recent endeavors in merging eye-tracking technology with VR herald new pathways for emotion research, although they come with their sets of challenges (Geraets et al., 2021; Gori et al., 2021; Tabbaa et al., 2021).

In synthesizing the above, it becomes evident that the symbiotic evolution of VR and Emotion Recognition technologies offers a tantalizing promise - a deeper, more nuanced understanding of human emotions, and myriad ways to explore, interact, and harness these emotions in previously uncharted terrains.

## 2. VR emotion detection system

### 2.1. Design of the System Architecture

**Figure 1:**
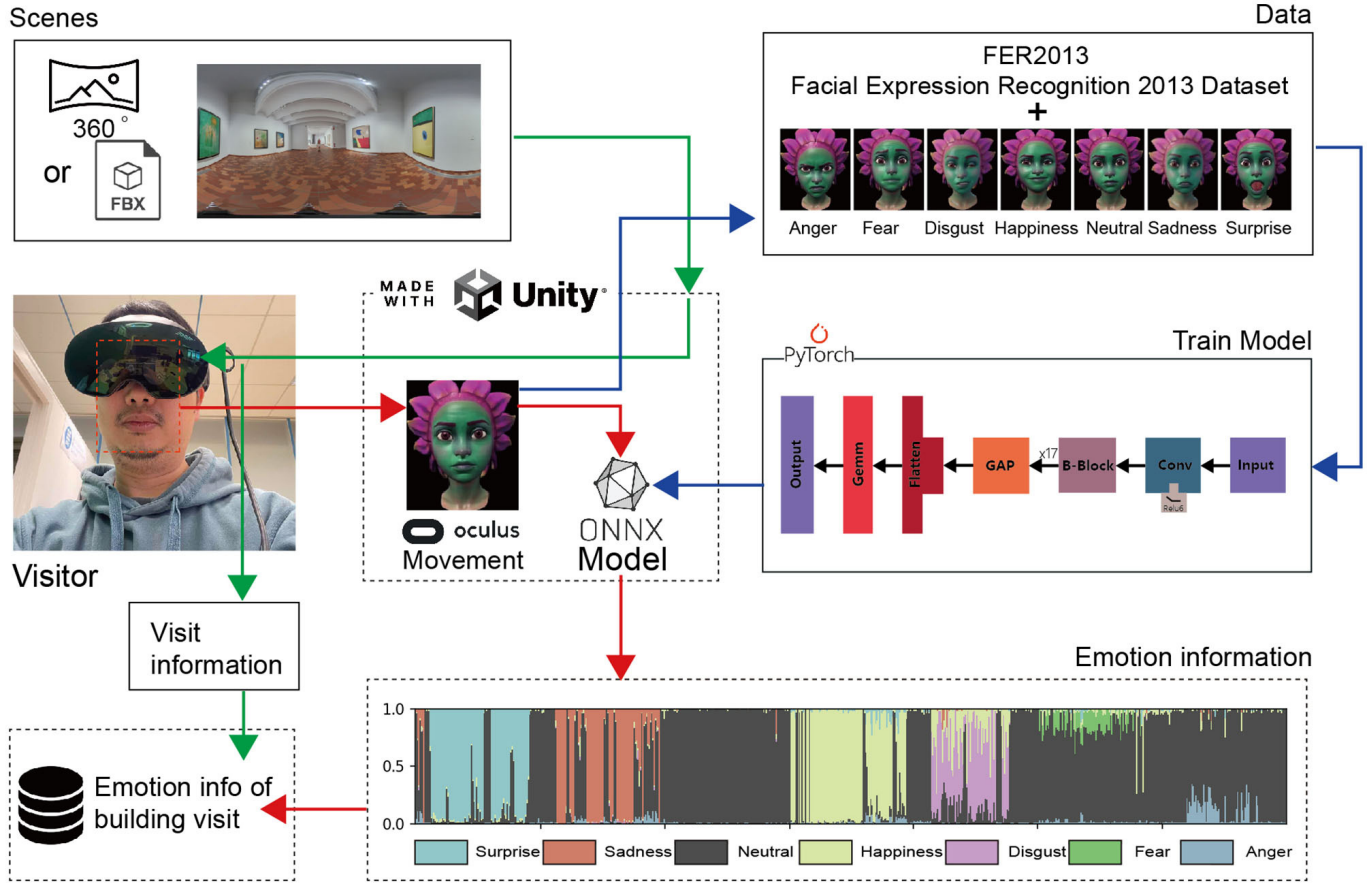
Design of the system architecture.

In constructing a VR-based emotion recognition system (see Figure 1), we adopted a comprehensive system architecture and suite of software tools to ensure precise, efficient emotion detection alongside an immersive virtual reality experience. Firstly, we chose Meta Quest Pro as the principal input device. This decision was rooted in Quest Pro's capabilities, as it not only supports conventional VR inputs but also incorporates natural facial expression recognition sensors. While similar devices, such as the Vive, also feature facial tracking capabilities that can be paired with VIVE Focus 3 to track facial expressions (Hu et al., 2023), Vive's software programming isn't based on Unity. This makes it inconvenient for potential transitions to Apple's forthcoming Vision Pro (Waisberg et al., 2023), which boasts superior facial recognition capabilities. Thus, our choice veered towards Quest Pro. By capturing users' facial movements, Quest Pro can map their real-world expressions to virtual characters within the software. Even though we analyzed facial imagery and eye-tracking capabilities, the use of virtual avatars ensured that no personal information was retained, thereby guaranteeing privacy and security.

For rendering the virtual environment, we opted for Unity as the rendering engine. Its robust compatibility with Meta Quest Pro and extensive SDK support allowed us to rapidly design and deploy realistic virtual scenarios. Opting for Unity not only streamlined the developmental process but also provided a powerful graphics and physics engine (Meta, 2023). This support underpins a myriad of intricate virtual interactions and visual effects. In terms of environmental inputs, our software was designed to allow users to choose their scenes, offering flexibility for other researchers to set the scenarios they wish to study.

For facial emotion analysis, we decided on PyTorch for deep learning. We combined virtual character data for seven fundamental emotions (surprise, sadness, neutral, happiness, disgust, fear, and anger) with the FER2013 database to create our dataset (Giannopoulos et al., 2018). Machine learning training yielded a pth model, which we subsequently converted to the ONNX model format using PyTorch. Unity's Barracuda package enabled us to use the Onnx model for inference and integrate it within the Quest Pro application. This accomplished the machine learning inference component within Unity, providing us with the desired emotion values.

Regarding data storage and management, the identified emotion data–collected at a frequency of 10 times per second–and the visitor's scene information were stored in an Excel spreadsheet. This method ensured timely and orderly data storage, making subsequent analysis and processing more convenient. Our focus on data storage underlines our holistic consideration of the entire system workflow, from emotion capture and analysis to virtual environment rendering and data storage.

### 2.2. Technical Details of Facial Recognition in Unity

**Figure 2:**
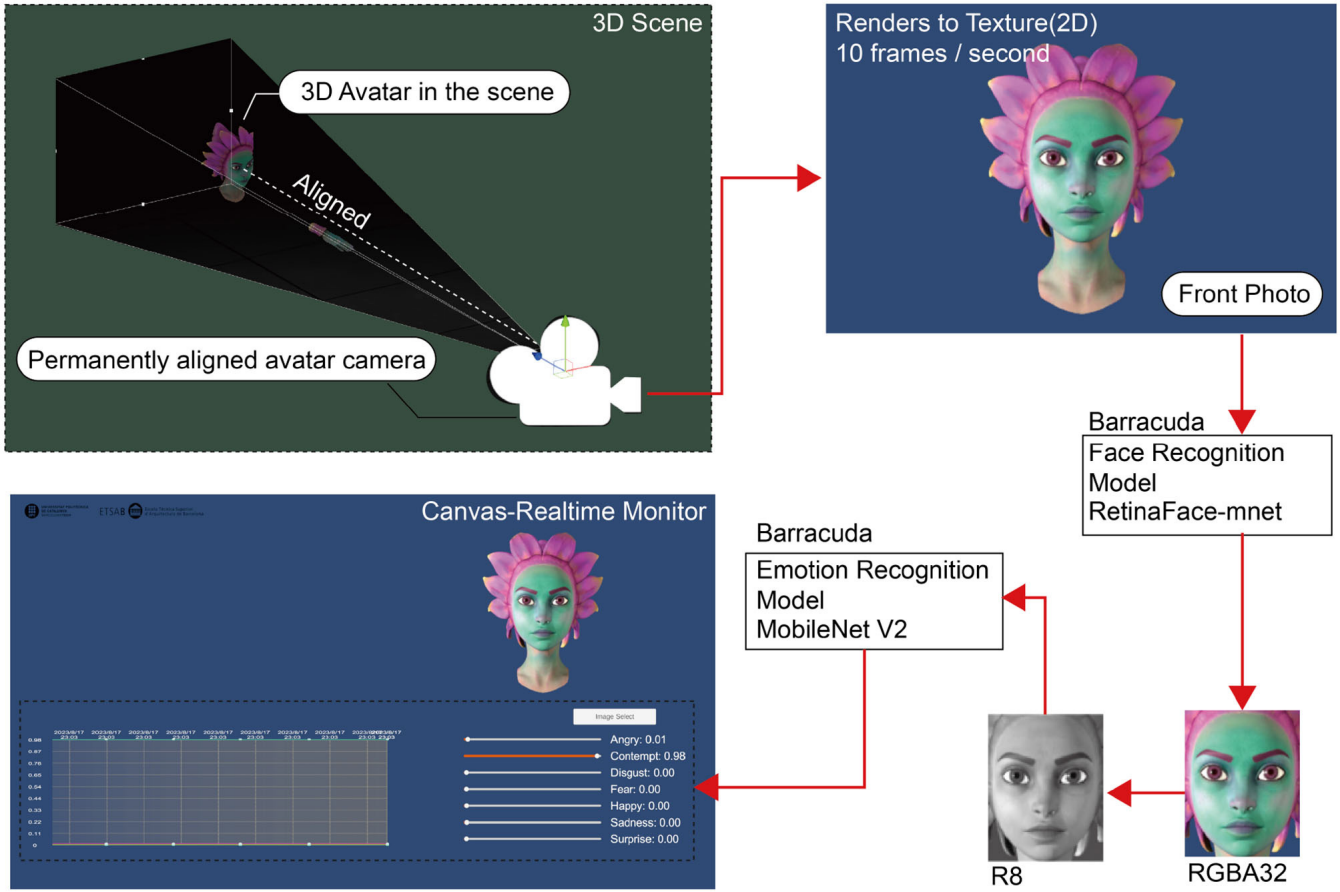
Technical details of facial recognition in unity.

Within the Unity environment (see Figure 2), we initially position the 3D Avatar into the desired scene. Researchers can select their own scenes using panoramic images or FBX models. To ensure real-time capture of the avatar's facial expressions, we placed a camera directly in front of the Avatar. This camera is programmatically set to always track and aim at the Avatar's face, capturing its frontal facial features from the optimal angle.

For real-time monitoring of the Avatar's facial expressions, we set the camera to render at a rate of 10 frames per second. This provides us with 10 2D texture images within a second, recording the Avatar's current facial state. Each generated texture is first fed into the retinaface-mnet model (Li et al., 2020). We chose this model because it's optimized specifically for portable devices. After being processed by the retinaface-mnet model, we can quickly and precisely extract the facial part from the original 2D texture image.

The retrieved facial texture then goes through a series of preprocessing steps, ensuring it's ready for further machine learning handling. These steps include cropping the image from its center to produce a 224x224 square image, scaling the image using bilinear interpolation, and converting the RGB image into grayscale. We opted for single-channel processing to avoid inconsistencies across different software and to improve efficiency. The processed R8 single-channel image is then sent to our pre-trained MobileNet V2 model (Sandler et al., 2018). This model performs real-time inferences on the image, identifying facial emotional characteristics accurately. Ultimately, this emotional data is rendered in real-time on a Canvas, allowing researchers to view and analyze the results directly on a monitor.

Our decision to adopt the above workflow was based on two main considerations. First, although we could directly process the raw data from the Quest Pro sensors, this would necessitate handling intricate micro-expression restorations. Given Meta's expertise in this domain, we decided to utilize their provided SDK as it is more reliable. Secondly, we wanted to ensure our solution is feasible across a variety of devices, such as the upcoming Apple Vision Pro. With potential differences in sensor technologies across devices, we opted to perform facial recognition directly on 2D images, bypassing the handling of raw sensor data. This choice ensures the versatility of our solution, allowing it to easily adapt to various devices.

### 2.3. Model selection: mobileNet V2

**Figure 3:**
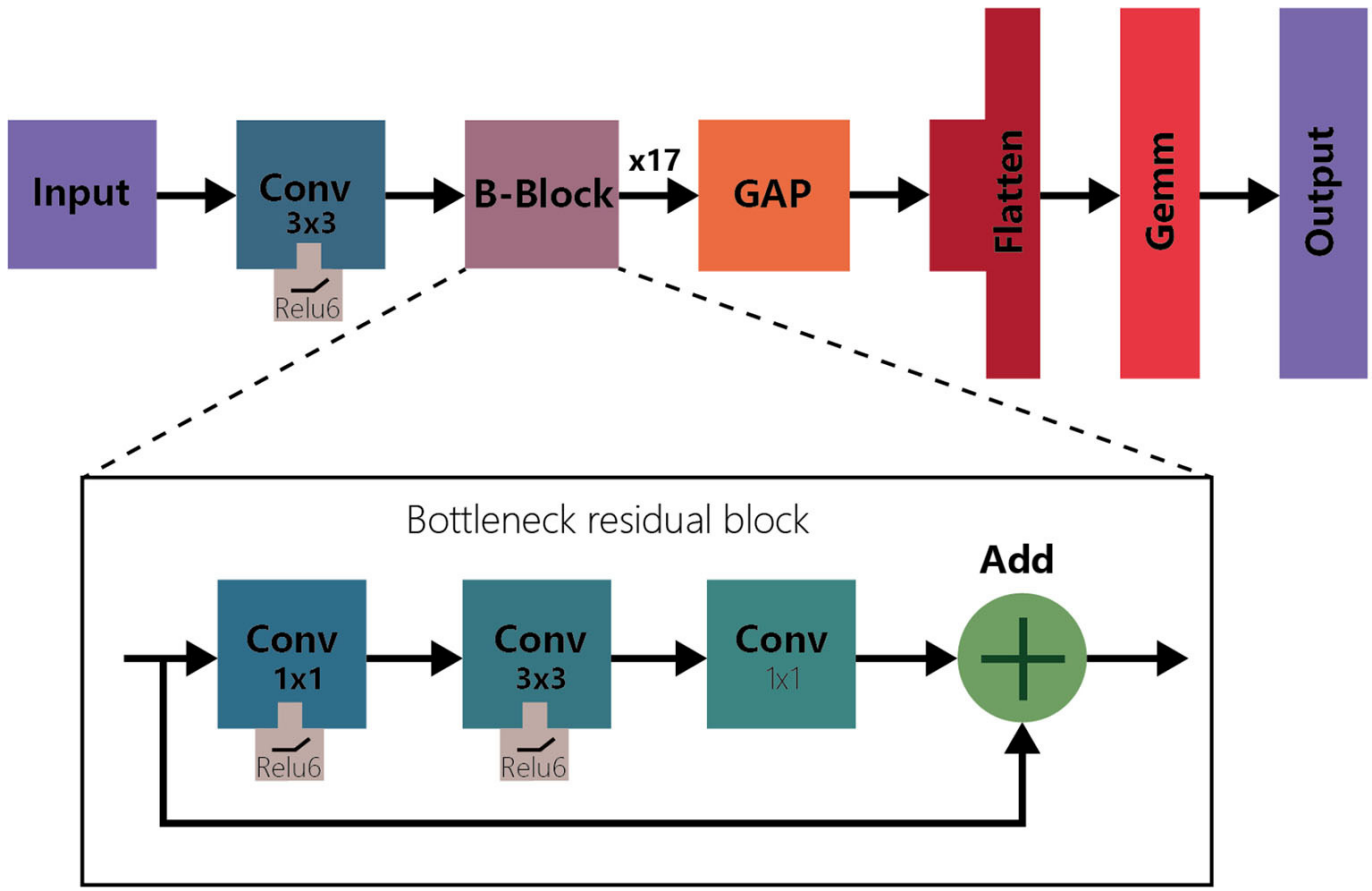
Architecture of MobileNet V2.

Choosing the right model is a crucial task in our facial expression recognition project. Initially, we tried deep learning models like ResNet 50 and VGG 19. However, due to compatibility issues with Barracuda, we ultimately settled on MobileNet V2 (see Figure 3) as our solution. MobileNet V2 is a lightweight convolutional neural network, especially suitable for running on resource-constrained devices like VR headsets. MobileNet V2 introduces what's called Inverted Residual Blocks, which utilize lightweight depthwise convolutions and pointwise convolutions. These inverted residual structures effectively reduce the computational complexity and the number of parameters of the model. Each inverted residual block in the model ends with a Linear Bottleneck structure, which helps minimize the model size and complexity while retaining essential information. This structural optimization allows MobileNet V2 to achieve optimizations in speed and performance while maintaining a high sensitivity to complex facial expression features. Compared to other larger and more intricate models, MobileNet V2 boasts a smaller footprint and faster computation speed, making it an ideal choice for real-time analysis and tracking of user facial expressions in virtual environments. Furthermore, due to the efficient performance of the MobileNet V2 model, it also allows us to achieve seamless real-time emotional mapping on VR devices, providing users with a more natural and immersive virtual reality experience.

## 3. Method

### 3.1. Participants

In our facial expression recognition experiment, we had a total of 15 participants, all of whom came from a university in a major city, with ages ranging from 19 to 42. All participants were university students, covering a variety of academic backgrounds. We did not record the gender information of the participants because the focus of the experiment was on facial expression recognition, and all participants' facial expressions were mapped onto the same avatar. Given that the aim of the experiment was to test the feasibility of VR facial expression recognition, gender was not a primary consideration. Furthermore, not recording gender also adhered to some ethical principles of global health research (Heidari et al., 2016). Participants with myopia were allowed to wear corrective glasses during the experiment. All participants were informed and signed a consent form, ensuring the experiment met ethical standards.

### 3.2. Experiment procedure

Once the participants understood the study's objectives and gave their consent, they were assisted in equipping and calibrating the VR tools. This ensured both comfort for the participant and accurate detection for the study. The procedure was primarily categorized into two phases: the emotion demonstration and the experiment execution. During the emotion demonstration phase, participants were introduced to standardized expressions for seven primary emotions: surprise, sadness, neutral, happiness, disgust, fear, and anger, presented in this specific sequence. Participants were then guided to mimic these emotions in the same order.

In the execution phase, at every minute interval, participants were prompted to emulate the specified emotions within the VR setting. Concurrently, computer monitors displayed and chronicled their avatar's facial expressions, adhering to the previously mentioned emotion sequence. After the experiment, thorough disinfection of the VR equipment was carried out. This ensured both the hygiene of the next participant and the undisturbed functioning of subsequent sessions. As a precautionary measure against interfering with the equipment's sensors, participants were advised against wearing disposable eye masks.

### 3.3. Data analysis

For every emotion presented in the sequence – surprise, sadness, neutral, happiness, disgust, fear, and anger – we extracted a 10-second data segment from each participant's feedback. Although participants were instructed to express each emotion within a minute, inconsistencies in their response time and the subtle nuances of facial expressions necessitated that we focus on a 10-second window, deeming it sufficient to capture the core of each emotion.

Rather than directly averaging the values within this time frame, we honed in on peak emotion intensity. Given our system's capability of capturing emotion at 10 frames per second, this 10-second segment provided 100 frames of data. From these, we pinpointed the top 10 emotion values, effectively representing the most intense emotion readings in a one-second span, though not necessarily from a consecutive second. This approach ensured our evaluations were centered on the most pronounced expression of each emotion, minimizing the influence of subdued or transitional values.

Once the top 10 values for each emotion from every participant were aggregated, we derived an average across all 15 participants. This methodology resulted in our confusion matrix, a detailed portrayal of the system's efficacy in discerning each emotion. In Table 1, the horizontal 'Emotion Requested of Participant' row indicates the emotions participants were instructed to show. The vertical 'Emotion' column represents the system's detected expressions. For instance, the 88.66% under 'Surprise' against the 'Surprise' row means that our system accurately identified the 'Surprise' expression 88.66% of the time when it was presented.

**Table 1:** Accuracy percentages of the VR facial expression recognition system. The table shows the system's detection rate for each emotion, in comparison to the emotion that participants were instructed to express.Each column sums up to 100% indicating the recognition rate of predicted emotions, while the sum of values in rows may vary.

	**Emotion requested of participant**						
**Emotion**	**Surprise**	**Sadness**	**Neutral**	**Happiness**	**Disgust**	**Fear**	**Anger**
Surprise	88.66%	0.00%	0.20%	0.14%	0.25%	1.46%	1.02%
Sadness	4.98%	93.11%	0.02%	0.00%	0.87%	0.06%	1.17%
Neutral	2.78%	0.49%	97.52%	3.87%	18.83%	68.41%	70.50%
Happiness	2.72%	5.20%	0.23%	94.15%	8.21%	6.35%	2.34%
Disgust	0.00%	0.15%	0.00%	0.00%	71.24%	4.75%	0.00%
Fear	0.00%	0.00%	0.00%	1.17%	0.00%	16.48%	0.00%

## 4. Result

In our VR emotion recognition experiment, we utilized a confusion matrix to evaluate the system's ability to identify various emotions(see Table 1). The results indicate that specific emotions such as “Neutral”, “Happiness”, “Sadness”, and “Surprise” achieved relatively high accurate identification rates. Particularly, the identification rate for “Neutral” reached a remarkable 97.52%, suggesting that the model can distinguish this emotion very accurately. The recognition rate for the “Happiness” emotion reached 94.15%, while “Sadness” and “Surprise” were at 93.11% and 88.66%, respectively. However, for emotions like “Anger” and “Fear”, the model's performance was relatively unsatisfactory. Especially for the “Anger” emotion, its accurate rate was only 24.84%, and it was frequently misidentified as “Neutral” with an error rate as high as 70.50%. Similarly, the “Fear” emotion was often misclassified as “Neutral”, with an error rate reaching 68.41

Regarding other emotions, such as “Disgust”, although its recognition rate was 71.24%, it was sometimes confused with 'Neutral', having an error rate of 18.83%. For the “Happiness” emotion, despite its high accuracy rate, there was still confusion with “Disgust” and “Sadness”, with error rates of 8.21% and 5.20% respectively. Considering the results as a whole, it's evident that the model displayed clear strengths and weaknesses in recognizing certain emotions. Particularly concerning is the model's difficulty in identifying the “Anger” and “Fear” emotions. As observed from our matrix (Table 1), certain rows have notably high recognition rates. This indicates that other true emotions are frequently misclassified into the respective predicted emotion categories. Such insights shed light on areas where the model might require refinement

## 5. Discussion

In this research, we delved deeply into VR emotional recognition, aiming to uncover the potential capabilities and limitations of the system. Our findings underscore the complexity of emotion recognition and highlight the challenges of current technology and models.

### 5.1. Model recognition robustness and limitations

**Figure 4:**
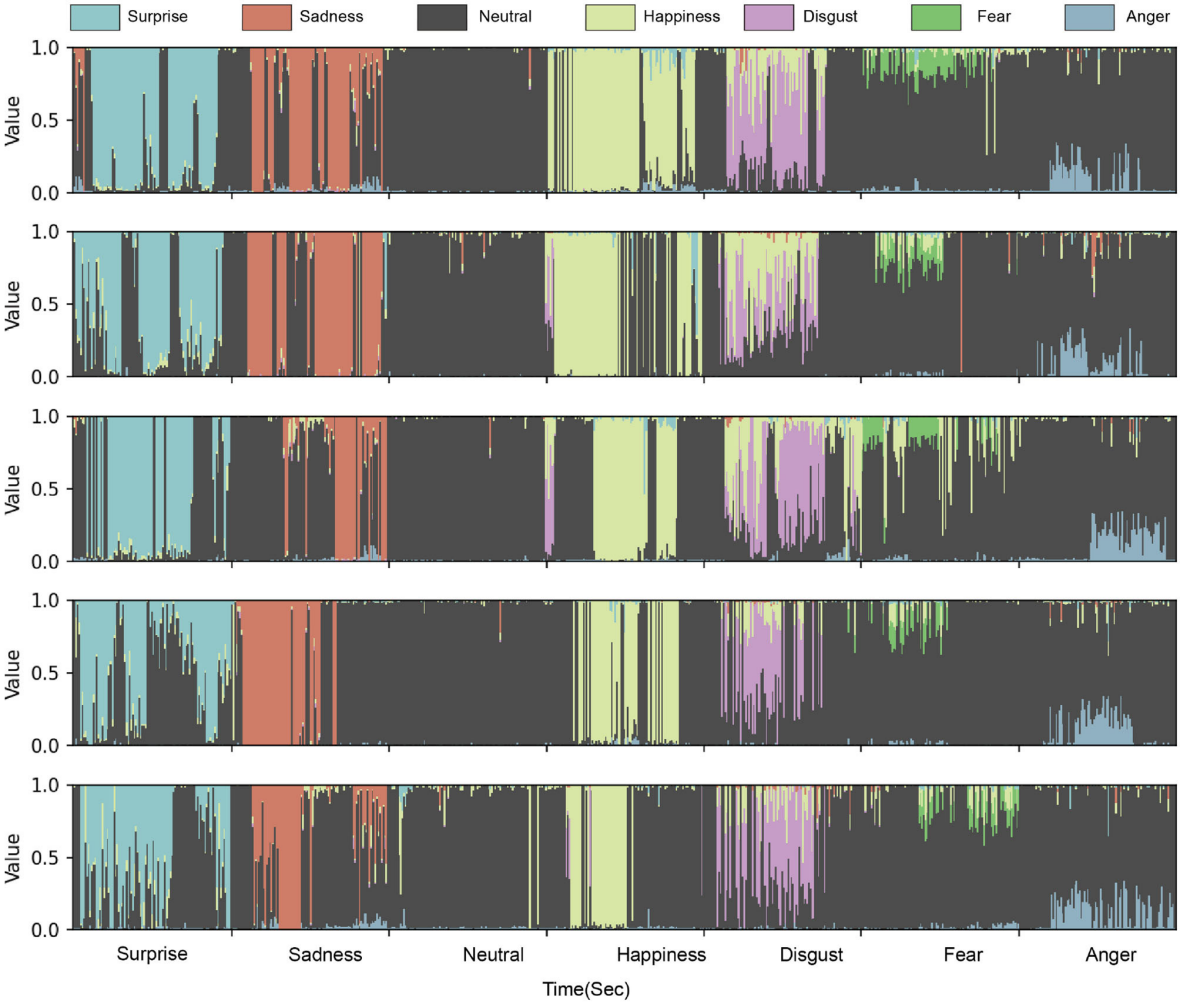
This figure displays the timeline of emotions expressed by the participants, represented on the X-axis, following the sequence: surprise, sadness, neutral, happiness, disgust, fear, and anger. The Y-axis indicates the recognition rate of these emotions, ranging from 0 to 1. Notably, within each of the seven emotional features, the black values specifically represent the recognition rates for the “neutral” emotion.

The high recognition rates of specific emotions, such as “Neutral”, “Happiness”, “Sadness”, and “Surprise”, point out the model's superiority in certain aspects, particularly those emotions that may have clear physiological or facial markers. However, for emotions like “Anger” and “Fear”, the model's performance declined (see Figure 4). This could be due to the overlapping physiological and facial features of these emotions with others, combined with a lack of samples for these emotions during model training, leading to its deficiencies. As observed from our matrix (Table 1), certain rows (specifically, 'Neutral') have notably high recognition rates. This indicates that other true emotions are frequently misclassified into the respective predicted emotion categories. Such insights shed light on areas where the model might require refinement.

### 5.2. Impact of device choice

Results obtained from the Quest Pro device indicate that certain emotions, especially “Anger” and “Fear”, might be challenging to capture accurately due to the precision of VR devices. In contrast, the Vision Pro, with its higher precision and additional sensors, could potentially offer more accurate emotion recognition outcomes. Nonetheless, our current research has established a solid foundation for future endeavors.

### 5.3. Comparing eye-tracking technology

In recent years, although VR eye-tracking technology has been explored in emotion recognition, solely relying on it has inherent limitations. The complexity and diversity of emotions imply that single eye-tracking might be insufficient to capture all emotional nuances (Geraets et al., 2021; Gori et al., 2021; Tabbaa et al., 2021). For instance, different emotions could lead to similar eye behaviors; anger and anxiety might both manifest the same eye fixations, yet they are physiologically and psychologically distinct. Moreover, emotions are not solely reflected in facial expressions; subtle changes in head movements and body trajectories could also reveal nuanced emotional differences. Therefore, a more holistic approach, integrating eye, face, and motion trajectory recognition, is necessary.

### 5.4. Future directions

We recommend using more advanced devices and delving deeper into the physiological responses of emotions like “Anger” and “Fear”, considering the integration of VR device motion trajectories with facial recognition. By combining more sophisticated technologies and larger datasets, there's immense potential and room for improvement in this field.

Overall, this study highlights the challenges and opportunities in the domain of VR emotion recognition. While current models and technologies still have their limitations, with the continual advancement of technology, VR facial emotion recognition is bound to find broad applications in the field of psychological research.

## Data availability statement

The raw data supporting the conclusions of this article will be made available by the authors, without undue reservation.

## Ethics statement

The studies involving humans were approved by Ethics Committee of the Universitat Politcnica de Catalunya. The studies were conducted in accordance with the local legislation and institutional requirements. The participants provided their written informed consent to participate in this study. Written informed consent was obtained from the individual(s) for the publication of any identifiable images or data included in this article.

## Author contributions

ZZ: Conceptualization, Data curation, Methodology, Software, Visualization, Writing—original draft. JF: Methodology, Supervision, Writing—review and editing. LG: Methodology, Supervision, Writing—review and editing.
